# Co-Endemic Zoonoses, Divergent Dynamics: A Comparative Spatiotemporal Analysis of Crimean–Congo Hemorrhagic Fever and Brucellosis with Implications for Integrated Surveillance

**DOI:** 10.3390/tropicalmed11090255

**Published:** 2026-09-09

**Authors:** Serdar Karakullukçu, İrem Dilaver, Murat Topbaş, Merve Erdoğan, Hüsniye Ebru Çolak, Meral Fidan Uçan

**Affiliations:** 1 Department of Public Health, Faculty of Medicine, Karadeniz Technical University, Trabzon 61080, Türkiye; iremhekimoglu@ktu.edu.tr (İ.D.); mtopbas@ktu.edu.tr (M.T.); m.erdogan118@gmail.com (M.E.); 2 Department of Geomatics Engineering, Faculty of Engineering, Karadeniz Technical University, Trabzon 61080, Türkiye; ecolak@ktu.edu.tr; 3 Gümüşhane Provincial Health Directorate, Gümüşhane 29100, Türkiye; meral.fidanucan@saglik.gov.tr

**Keywords:** spatial epidemiology, geographic information systems, space-time clustering, zoonotic surveillance, livestock-associated zoonoses, One Health

## Abstract

Co-endemic zoonoses may occur in the same broad geographic settings but not necessarily the same communities or time periods. This study investigated whether Crimean–Congo hemorrhagic fever (CCHF) and brucellosis exhibit convergent or divergent spatiotemporal patterns across geographic scales in a livestock-dependent, CCHF-endemic province of Türkiye. This retrospective study analyzed confirmed human CCHF and brucellosis cases reported to the national infectious disease surveillance system between 2011 and 2024. Temporal analyses, kernel density estimation, settlement-level random labeling, and within- and cross-disease Knox tests were performed. Among the 611 eligible cases, CCHF (*n* = 442; 72.3%) and brucellosis (*n* = 116; 19.0%) accounted for 91.3%. CCHF was strongly seasonal, with 99.3% of cases occurring between April and September, whereas brucellosis occurred throughout the year. Although broad density surfaces partially overlapped, same-settlement co-occurrence was substantially lower than expected (O/E = 0.37; *p* < 0.001) and remained so in the sensitivity analysis (O/E = 0.74; *p* = 0.001). Cross-disease analysis showed no evidence that CCHF and brucellosis occurred closer together in both space and time than expected. Annualized CCHF concentration peaked in 2020–2021, whereas brucellosis peaked in 2022–2024. Integrated surveillance may use shared regional infrastructure, but interventions should be tailored to disease-specific settlements, transmission pathways, and periods of risk.

## 1. Introduction

Zoonotic diseases are major public health concerns that reflect the interconnectedness of human health, animal health, environmental conditions, and food systems. In livestock-dependent settings, multiple zoonoses may be endemic within the same population; however, their occurrence in the same geographic area does not necessarily mean that they affect the same communities, settlements, or periods. Differences in transmission routes, daily practices, population mobility, and seasonal environmental conditions may create disease-specific patterns of community exposure. Understanding these differences is essential for balancing the joint targeting of multiple zoonoses within a shared geographic area against disease-specific prevention [1,2].

Crimean–Congo hemorrhagic fever (CCHF) and brucellosis are two important zoonoses that occur in livestock-dependent communities but differ substantially in their transmission routes. While the risk of CCHF is most pronounced in livestock farmers, shepherds, agricultural workers, and people who come into contact with the blood or tissues of animals, people living in rural and semi-rural areas where infected ticks are found or who are in these environments due to outdoor activities are also at risk [3,4,5]. Unprotected contact with patients’ blood and body fluids has been demonstrated to pose a risk of secondary transmission to family members and healthcare workers. In contrast, brucellosis is transmitted through direct contact with infected animals and their reproductive materials, as well as through the consumption of unpasteurized milk and dairy products [6,7]. The distribution of animal products to urban centers via family, market, and trade networks has the potential to extend the risk of brucellosis to individuals who have no direct contact with animals and beyond the production area. Thus, community patterns of CCHF may reflect tick ecology, outdoor exposure, and seasonal activities, whereas brucellosis may also reflect food production, distribution, and consumption practices.

CCHF and brucellosis may affect similar occupational and social groups and share certain clinical and laboratory features. The detection of brucellosis alone or as a co-infection among patients evaluated for suspected CCHF demonstrates the need to consider both diseases in the differential diagnosis in endemic settings [8]. However, clinical coexistence does not necessarily indicate that the diseases cluster in the same communities or during the same periods. Addressing this population-level question requires comparison of their spatial and temporal distributions beyond individual clinical encounters. Geographic overlap and temporal concordance cannot be inferred from provincial case counts or incidence rates alone.

Geographic information systems (GISs) provide an appropriate epidemiological framework for this comparison by integrating surveillance data with spatial and temporal information [9,10]. Mapping case distributions and comparing density surfaces can reveal whether diseases share broad geographic areas of concentration, while temporal and space–time analyses can assess whether geographic overlap is accompanied by similar temporal patterns. However, kernel density surfaces smooth cases within a specified bandwidth and cannot, by themselves, establish whether cases within the same density area originate from the same settlement. Broad-scale overlap should therefore be evaluated separately from settlement-level co-location.

The geographic distribution of CCHF in Türkiye has been investigated in relation to tick ecology, climate, land use, habitat fragmentation, and livestock-related environmental conditions, and several spatial risk models have been developed [11,12,13,14]. Spatial studies of human brucellosis have similarly identified regional clustering and associations with livestock-related indicators [15]. However, most previous studies have examined these diseases separately, and direct comparisons of their spatial overlap and temporal variation within the same surveillance system and social environment remain limited.

Located in northeastern Türkiye, Gümüşhane provides an informative eco-epidemiological setting for investigating these contrasting zoonotic dynamics. The province lies within an established CCHF-endemic region [5,11,12,13,14] and has a strong socioeconomic reliance on traditional highland livestock farming [16,17], creating a setting in which both CCHF- and brucellosis-related exposures may occur within the same regional population. Its mountainous terrain, dispersed rural settlements, and close connections between livestock-dependent rural communities and district centers further provide an appropriate context for comparing zoonoses with distinct transmission pathways. Studying both diseases within the same population and surveillance framework also reduces variation arising from differences in data sources, surveillance structures, and regional context. Therefore, Gümüşhane represents a highly informative setting for assessing whether broad geographic co-endemicity translates into shared settlement-level spatial and temporal patterns.

This study aims to determine whether human cases of CCHF and brucellosis, despite being co-endemic within the same livestock-dependent region, exhibit convergent or divergent spatial and temporal patterns across geographic scales, and to assess the implications of these patterns for integrated zoonotic disease surveillance.

## 2. Materials and Methods

### 2.1. Study Design and Area

**Figure 1:**
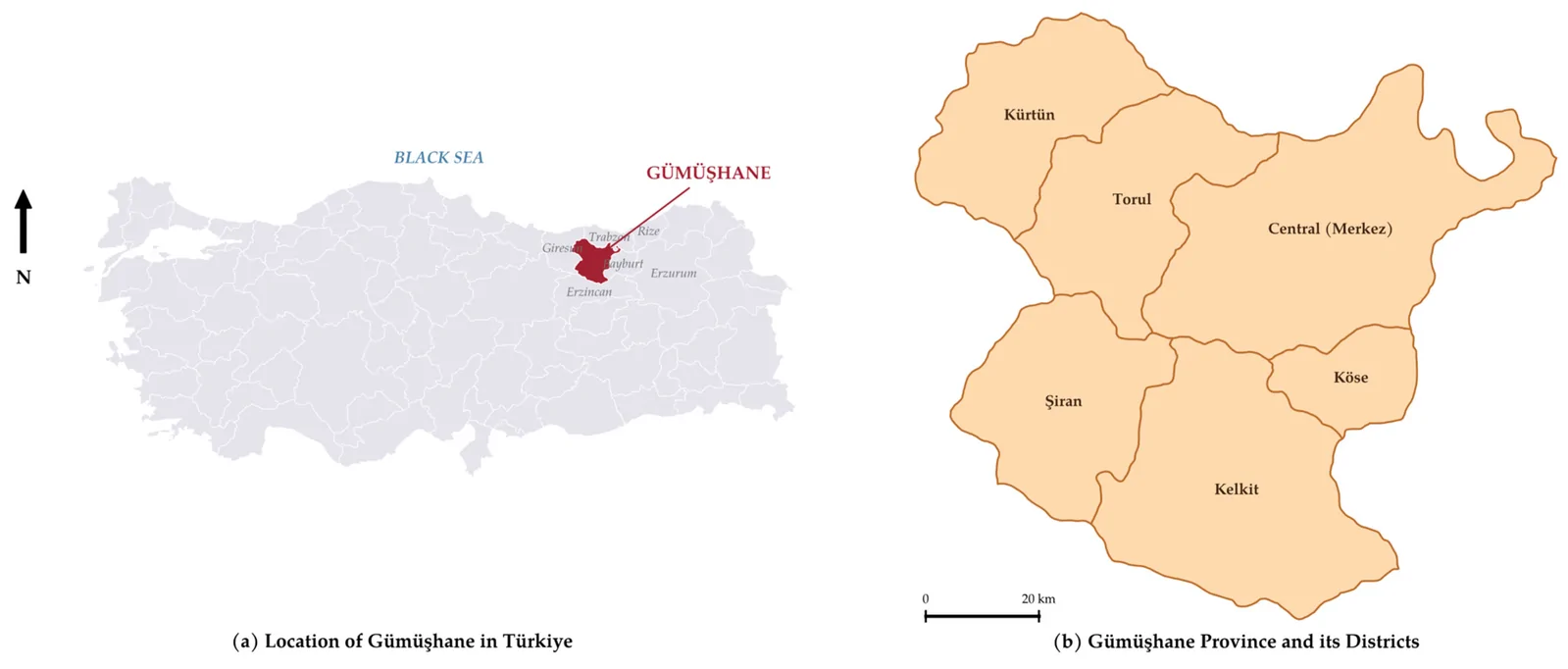
Location map of the study area: (**a**) position of Gümüşhane Province within Türkiye; (**b**) Gümüşhane Province and its districts.

This retrospective surveillance-based spatial epidemiology study included zoonotic disease cases reported in Gümüşhane Province between 1 January 2011 and 31 December 2024. Gümüşhane is located in the Eastern Black Sea Region of Türkiye and comprises six districts: Central (Merkez; approximately 40.459° N, 39.483° E), Kelkit (40.128° N, 39.433° E), Köse (40.208° N, 39.655° E), Kürtün (40.702° N, 39.086° E), Şiran (40.190° N, 39.126° E), and Torul (40.558° N, 39.293° E) (Figure 1). The province is characterized by mountainous terrain, highland livestock farming, and dispersed rural settlements [16].

### 2.2. Study Population and Data Source

The study population comprised human cases residing in Gümüşhane Province during the study period who were reported as confirmed cases of a notifiable zoonotic disease. Zoonotic diseases are statutorily notifiable in Türkiye, and cases are reported to the national Infectious Disease Surveillance and Early Warning System operated by the Turkish Ministry of Health. Case data were obtained from the records of the Infectious Diseases Unit of the Gümüşhane Provincial Health Directorate, which maintains provincial notifications submitted to the national surveillance system; a staff member of the Provincial Health Directorate was among the co-authors of this study. An anonymized extract covering all zoonotic disease cases reported during the study period was obtained from these records. For each case, the available variables included age, sex, date of diagnosis, diagnosed disease, and residential address at the district and village/neighborhood levels. No personal identifiers were included in the dataset. Symptom-onset dates were not consistently available in the surveillance records; therefore, temporal analyses were based on the recorded date of diagnosis. The available surveillance records did not allow for differentiation between laboratory confirmation date and notification date; therefore, diagnosis date was used as the most consistently available temporal variable for both diseases.

The temporal parameters of the study were delineated by the year, month, and season of diagnosis. The seasons were classified into the following categories: spring (March–May), summer (June–August), fall (September–November), and winter (December–February).

All cases had already been classified as confirmed in accordance with the national case definitions in force at the time of diagnosis. No diagnostic testing was performed as part of the present study, and the specific diagnostic assay used for each individual case was not available in the surveillance extract. Because the study covered the period from 2011 to 2024, diagnostic methods may have varied over time according to the national guidelines applicable during the corresponding period. Accordingly, the present analysis relied on the confirmed case classification recorded in the surveillance system rather than on any single diagnostic testing protocol.

The overall zoonotic disease profile included all 611 records reported during 2011–2024. Disease-specific analyses focused on CCHF (*n* = 442) and brucellosis (*n* = 116), which together accounted for 91.3% of the recorded zoonotic disease burden. All available cases were retained in the overall disease profile and non-spatial temporal analyses. Two brucellosis cases, one recorded in 2017 and the other in 2023, lacked usable settlement coordinates. These cases were retained in descriptive, temporal, and district-level analyses because their diagnosis dates and district information were available, but they were excluded from analyses requiring settlement coordinates. Direct spatial and spatiotemporal comparisons between CCHF and brucellosis were restricted to the common observation period of 2017–2024 because only two brucellosis cases were recorded before 2017, both in 2015. The common-period descriptive comparison comprised 256 CCHF cases and 114 brucellosis cases. After excluding the two brucellosis cases without usable settlement coordinates, coordinate-based comparisons included 256 CCHF cases and 112 brucellosis cases.

### 2.3. Geocoding

Disease and settlement names were first standardized, and date, age, sex, and address data were examined for internal consistency. Residential addresses were geocoded at the village or neighborhood level rather than at the individual household level. For each recorded settlement, settlement-center coordinates were obtained manually from Google Maps by searching the settlement name together with its recorded district and province (Gümüşhane), and the coordinates of the corresponding village or neighborhood center were retained in decimal degrees (WGS 84). This procedure yielded coordinates for 165 distinct CCHF settlements and 66 distinct brucellosis settlements, corresponding to 220 unique district–settlement combinations overall because some settlements reported cases of both diseases. All 442 CCHF cases (100%) and 114 out of 116 brucellosis cases (98.3%) were successfully geocoded; two brucellosis records lacked sufficient address information and were excluded from coordinate-based analyses while being retained in descriptive, temporal, and district-level analyses.

Several consistency and verification steps were applied. Orthographic variants referring to the same settlement, such as spelling or spacing differences in the same village name, were reconciled using the recorded district information and the verified settlement-center coordinates. Conversely, settlements with distinct names that happened to be assigned identical or closely similar coordinates were retained as separate settlements after manual verification against their district and settlement names, thereby avoiding the erroneous merging of different villages into a single location. Where a settlement name could not be matched directly, for example, for very small hamlets or settlements sharing a name with another locality, an approximate location near the relevant district center was used. Such records were flagged as approximate and accounted for 14 out of the 556 geocoded cases (2.5%).

Geocoding accuracy was further assessed using a spatial validation procedure in which each geocoded point was compared with the boundary polygon of its recorded district. At the case level, 540 out of 556 geocoded cases (97.1%) fell within the polygon of their recorded district. The remaining 16 cases, representing nine settlements, were located close to the boundary of their recorded district (median distance, 1.4 km; maximum, 3.6 km). Manual review confirmed consistency between the recorded district, settlement identity, and assigned coordinates. These discrepancies were considered consistent with limitations in the resolution and alignment of the administrative boundary layer used for validation rather than with clear geocoding errors. Because district-level analyses relied on the district recorded in the surveillance data rather than on the validation polygons, these boundary discrepancies did not affect the district-level results. Multiple cases from the same settlement were assigned the same settlement-center coordinates but were retained as separate case records in the analyses. Accordingly, mapped points represent village or neighborhood locations rather than individual residences or places of exposure. All spatial data used in the distance- and density-based analyses were transformed to the WGS 84/UTM Zone 37N coordinate reference system (EPSG:32637).

### 2.4. Population Data

Annual provincial and district populations were obtained from the Address-Based Population Registration System of the Turkish Statistical Institute. Annual notification rates were calculated using the corresponding annual population and expressed per 100,000 population.

Period notification rates were calculated by dividing the cumulative number of cases by the sum of the annual district populations during the corresponding period and were expressed per 100,000 person-years. District-level CCHF rates covered 2011–2024 and included all 442 cases. Because only two brucellosis cases were recorded before 2017, district-level brucellosis rates were calculated for 2017–2024 and included 114 cases. The two brucellosis cases without settlement coordinates were retained in district-level rate calculations because valid district information was available.

### 2.5. Statistical Analysis

Statistical analyses were performed using IBM SPSS Statistics version 27.0 (IBM Corp., Armonk, NY, USA). The distribution of continuous variables was assessed using graphical methods and the Kolmogorov–Smirnov test. Continuous variables with non-normal distributions are presented as median and interquartile range (IQR), and categorical variables as counts and percentages. CCHF and brucellosis were compared using the Mann–Whitney U test for continuous variables and the chi-square or Fisher’s exact test for categorical variables, as appropriate. Descriptive statistics were used to summarize the distribution of reported zoonotic diseases during the study period. Numbers and percentages were presented for each disease category. This analysis was descriptive; the selection of CCHF and brucellosis for advanced analyses was based on their contribution to the overall disease burden and the availability of sufficient case numbers. Monthly and seasonal case distributions were evaluated using chi-square goodness-of-fit tests, and the seasonal distributions of CCHF and brucellosis were compared using the chi-square test. Monotonic trends in annual case counts were evaluated using Kendall’s tau for CCHF during 2011–2024 and for brucellosis during 2017–2024. All tests were two-sided, and *p* < 0.05 was considered statistically significant.

### 2.6. Spatial Analysis

Spatial analyses were conducted using QGIS version 3.44.12 (QGIS Development Team) in the WGS 84/UTM Zone 37N coordinate reference system (EPSG:32637). Settlement-level case distributions were displayed using proportional symbols. Spatial co-occurrence comparisons between CCHF and brucellosis were restricted to the common observation period of 2017–2024 and included 256 CCHF and 112 brucellosis cases with usable coordinates.

Settlement urbanicity was determined using the 2020 Global Human Settlement Layer Settlement Model (GHS-SMOD) grid (R2023A; 1 km resolution) and its Degree of Urbanization (DEGURBA) classification [18]. Classes 21–30 were categorized as urban and classes 11–13 as rural. The primary analysis included 556 coordinate-assigned cases, of which 542 were directly matched to verified settlement coordinates and 14 were approximately matched after settlement-name standardization and district-level verification. A sensitivity analysis was restricted to the 542 directly matched records.

Separate overall kernel density estimation (KDE) surfaces were generated using all coordinate-available cases: 442 CCHF cases recorded during 2011–2024 and 114 brucellosis cases recorded during 2015–2024. KDE was performed using case-weighted settlement locations, a quartic kernel, a 10 km bandwidth, and a 500 m cell size. Surfaces were clipped to the provincial boundary, and sensitivity analyses used bandwidths of 5 and 15 km. KDE values were retained on their original output scale, with disease-specific color scales extending from zero to the maximum density in each surface. Accordingly, KDE maps represent case concentration rather than population-adjusted risk.

As a secondary exploratory analysis, annualized KDE surfaces were generated for both diseases to compare spatial concentration before, during, and after the main COVID-19 pandemic period. The periods were 2011–2019, 2020–2021, and 2022–2024 for CCHF, and 2017–2019, 2020–2021, and 2022–2024 for brucellosis. The two brucellosis cases recorded in 2015 were included in the overall KDE but excluded from the temporal comparison because consistent brucellosis reporting began in 2017. Case counts were divided by the number of years in each period before KDE estimation. A common color scale was applied across the three periods within each disease, whereas disease-specific scales were used for CCHF and brucellosis. These analyses were descriptive and were not designed to assess a causal effect of the pandemic.

Spatial co-occurrence between CCHF and brucellosis was assessed for the common observation period of 2017–2024 using a random-labeling test implemented in R version 4.4.3 with a custom script. The analysis included 256 CCHF cases and 112 brucellosis cases with usable settlement coordinates. Case locations were held fixed, and disease labels were randomly permuted 9999 times while preserving the observed number of cases for each disease. For each permutation, the number of cross-disease case pairs assigned to the same settlement was calculated. The expected number of pairs was defined as the mean count across the 9999 permutations. Lower-tail permutation *p*-values were used to assess whether same-settlement co-occurrence was less frequent than expected. To evaluate the influence of settlements with large case counts, the analysis was repeated after retaining a maximum of five cases for each settlement–disease combination. A fixed random seed of 2026 was used, and Monte Carlo *p*-values were calculated using the +1 correction. To assess whether the observed settlement-level separation was attributable to differences in settlement urbanicity between the two diseases, an additional conditioned random-labeling sensitivity analysis was performed. Disease labels were permuted separately within the urban and rural strata defined by the GHS-SMOD/DEGURBA classification, while case locations were held fixed. This procedure preserved the observed disease composition within each urbanicity stratum. As in the primary analysis, 9999 permutations were performed, and the observed number of same-settlement cross-disease pairs was compared with the permutation-based expected count using the observed-to-expected ratio and a lower-tail Monte Carlo *p*-value.

### 2.7. Spatiotemporal Analysis

Spatiotemporal interaction within each disease was assessed separately for CCHF and brucellosis using the Knox space–time interaction test, implemented in R version 4.4.3 with a custom script. The analyses included all 442 CCHF cases and the 114 brucellosis cases with usable settlement coordinates. Spatial thresholds of 5 and 10 km represented short-range and broader local interaction, while temporal thresholds of 30 and 60 days represented approximately one- and two-month periods. Pairs occurring exactly at the specified spatial or temporal thresholds were included. The spatial thresholds were informed by the observed settlement geometry of the study area rather than chosen arbitrarily. The median distance from each settlement to its nearest neighboring settlement was 2.8 km (interquartile range, 1.7–3.6 km); therefore, a 5 km threshold captures a local scale encompassing many nearest-neighbor relationships, whereas a 10 km threshold represents a broader local neighborhood. Both thresholds remained well below the overall spatial scale of the province (median inter-settlement distance, 36 km; maximum, 108 km), with only 2.2% and 6.9% of all settlement pairs falling within 5 km and 10 km, respectively, indicating that the analyses were focused on local rather than province-wide spatial correspondence. The temporal thresholds of 30 and 60 days were selected as nested one- and two-month windows for assessing short-term space–time correspondence at the community level. Because CCHF and brucellosis differ in their incubation periods and temporal clinical course, and because diagnosis date rather than symptom-onset date was available, these thresholds were treated as exploratory community-level scales rather than as estimates of a specific transmission interval. The use of two spatial and two temporal thresholds allowed the consistency of the findings across alternative scales to be assessed.

For each threshold combination, the observed number of case pairs meeting both the spatial and temporal criteria was compared with expected counts obtained by randomly permuting diagnosis dates among fixed case locations 9999 times. A fixed random seed of 2026 was used, and Monte Carlo *p*-values were calculated using the +1 correction. Results were reported as observed and expected pair counts, observed-to-expected ratios, and unadjusted upper-tail permutation *p*-values. Cases assigned to the same settlement were treated as having a spatial distance of zero. A sensitivity analysis excluding within-settlement pairs was conducted to assess the influence of identical settlement-center coordinates.

Cross-disease spatiotemporal correspondence was assessed for the common observation period of 2017–2024 using 256 CCHF cases and 112 brucellosis cases with usable settlement coordinates. All pairs consisting of one CCHF case and one brucellosis case were evaluated using the same spatial and temporal thresholds. Expected counts were obtained by independently permuting diagnosis dates among fixed locations within each disease 9999 times. Observed-to-expected ratios greater than one indicated more cross-disease pairs than expected, whereas ratios below one indicated fewer pairs. Upper-tail *p*-values assessed excess correspondence, and lower-tail *p*-values assessed fewer-than-expected pairs. These *p*-values were reported as unadjusted exploratory results. The Knox analyses assessed settlement-level spatiotemporal correspondence and did not identify the locations or causes of individual clusters.

## 3. Results

### 3.1. Provincial Zoonotic Disease Profile

A total of 611 confirmed zoonotic disease cases were included. CCHF accounted for 442 cases (72.3%) and brucellosis for 116 cases (19.0%); together, these two diseases represented 91.3% of all cases. The remaining cases comprised tularemia (*n* = 29), cystic echinococcosis (*n* = 20), Lyme disease (*n* = 2), and one case each of anthrax and leptospirosis (Table 1). Because CCHF and brucellosis dominated the recorded disease burden and provided sufficient case numbers, subsequent comparative spatial and spatiotemporal analyses focused on these two diseases.

### 3.2. Comparative Characteristics and Settlement-Level Urbanicity

The age distributions of CCHF and brucellosis cases were comparable (median: 49.0 years [IQR: 36.0–60.0] vs. 49.5 years [IQR: 38.0–59.0], respectively; *p* = 0.990). However, the proportion of male cases was higher for brucellosis than for CCHF (70.7% vs. 58.6%; *p* = 0.023).

Settlement-level urbanicity was evaluated for the 556 cases with available coordinates; two brucellosis cases without usable coordinates were excluded. Most cases of both diseases were classified as rural; however, urban classification was more frequent among brucellosis cases than among CCHF cases (22.8% vs. 15.2%); this difference approached but did not reach conventional statistical significance (*p* = 0.051). The direction of the difference was unchanged in the sensitivity analysis restricted to the 542 directly matched cases (23.9% vs. 14.5%), in which the difference was statistically significant (*p* = 0.019).

### 3.3. Temporal Patterns of CCHF and Brucellosis

**Figure 2:**
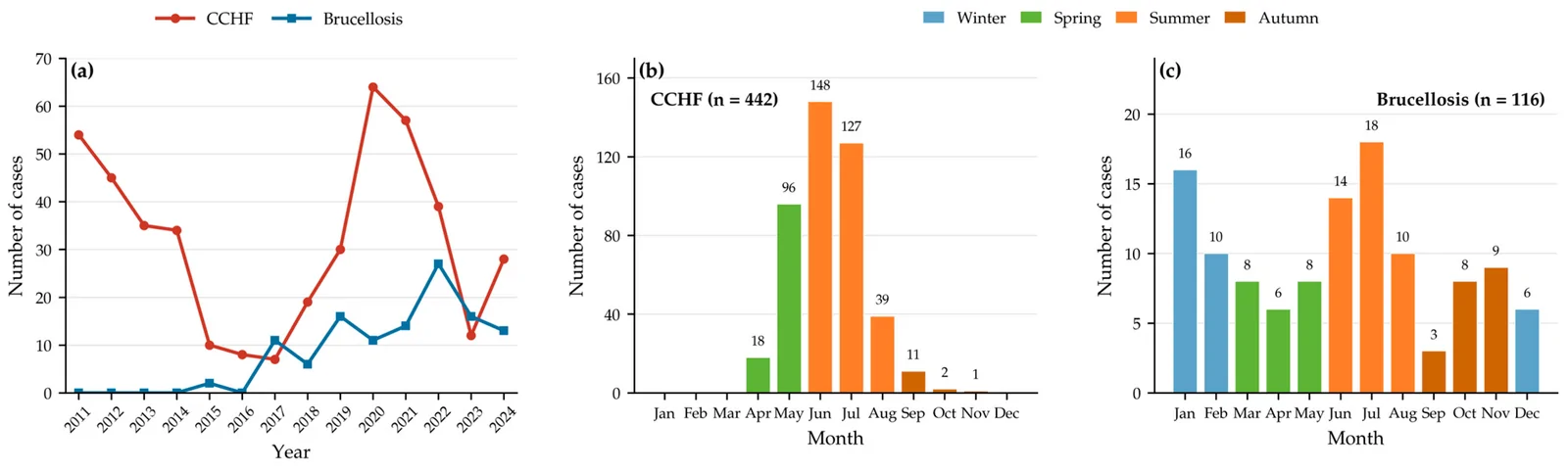
Temporal distribution of CCHF and brucellosis cases in Gümüşhane Province, 2011–2024: (**a**) annual case counts, (**b**) monthly distribution of CCHF cases, and (**c**) monthly distribution of brucellosis cases. Colors in panels (**b**,**c**) indicate seasons.

Annual CCHF case counts fluctuated throughout the study period, with the highest number recorded in 2020 (*n* = 64). No significant monotonic trend was detected between 2011 and 2024 (Kendall’s τ = −0.121, *p* = 0.591). Because only two brucellosis cases were recorded before 2017, its trend analysis was restricted to 2017–2024. Brucellosis cases peaked in 2022 (*n* = 27), with no significant monotonic trend during this period (Kendall’s τ = 0.371, *p* = 0.209; Figure 2a).

CCHF exhibited marked seasonality: 439 out of 442 cases (99.3%) occurred between April and September, with the highest numbers recorded in June (*n* = 148, 33.5%) and July (*n* = 127, 28.7%; Figure 2b). In contrast, brucellosis cases occurred throughout the year; July was the most frequent month (*n* = 18, 15.5%), and 59 out of 116 cases (50.9%) occurred between April and September (Figure 2c). Summer accounted for 71.0% of CCHF cases and 36.2% of brucellosis cases, whereas no CCHF cases occurred during winter.

The monthly and seasonal distributions differed significantly from uniform distributions for both CCHF (both *p* < 0.001) and brucellosis (*p* = 0.028 and *p* = 0.014, respectively). The seasonal distributions of CCHF and brucellosis also differed significantly (*p* < 0.001).

### 3.4. District-Level Distribution

District-level notification rates varied substantially between CCHF and brucellosis (Table 2). For CCHF, Kelkit recorded the highest number of cases (*n* = 185), whereas the highest notification rate was observed in Şiran (45.8 per 100,000 person-years). Şiran was followed by Kelkit (29.9), Torul (24.5), and Köse (21.7). The lowest CCHF rate was recorded in Kürtün (1.1 per 100,000 person-years).

For brucellosis, Kelkit accounted for 76 out of the 114 cases included in the 2017–2024 spatial analysis (66.7%) and had the highest notification rate (21.2 per 100,000 person-years). Rates in the remaining districts were substantially lower, ranging from 2.3 in Central to 8.4 in Torul. Thus, CCHF showed its greatest population-adjusted burden in Şiran, whereas brucellosis was predominantly concentrated in Kelkit.

### 3.5. Spatial Case Concentration and Temporal Changes in CCHF and Brucellosis

**Figure 3:**
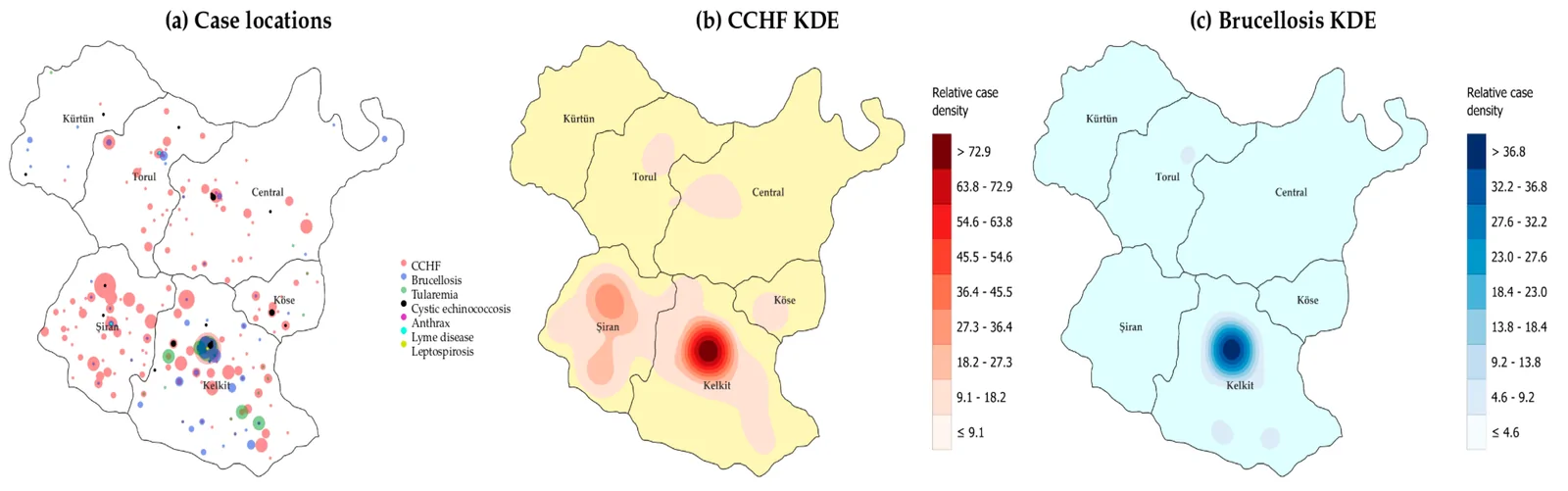
Spatial distribution and kernel density patterns of zoonotic disease cases in Gümüşhane Province. (**a**) Point distribution of coordinate-available zoonotic disease cases during 2011–2024; (**b**) kernel density surface of 442 CCHF cases recorded during 2011–2024; and (**c**) kernel density surface of 114 coordinate-available brucellosis cases recorded during 2015–2024. KDE surfaces were generated using a quartic kernel, a 10 km bandwidth, and a 500 m cell size. Disease-specific density scales were used in panels (**b**,**c**) and should be interpreted independently. The maps represent case concentration rather than population-adjusted risk.

The point distribution of coordinate-available zoonotic disease cases demonstrated that cases were distributed across the province but were more frequently observed in its southern districts (Figure 3a). CCHF and brucellosis constituted most of the mapped cases, whereas the other zoonoses occurred less frequently and were more dispersed.

The disease-specific KDE surfaces showed partially overlapping but distinct patterns of case concentration (Figure 3b,c). CCHF exhibited a relatively broad concentration across the southern part of the province, with the principal high-density area in Kelkit and additional concentrations in Şiran (Figure 3b). Brucellosis showed a more localized pattern, with its most prominent concentration also occurring in Kelkit (Figure 3c). Thus, although the two diseases shared a broad concentration area around Kelkit, their overall density patterns were not spatially identical.

**Figure 4:**
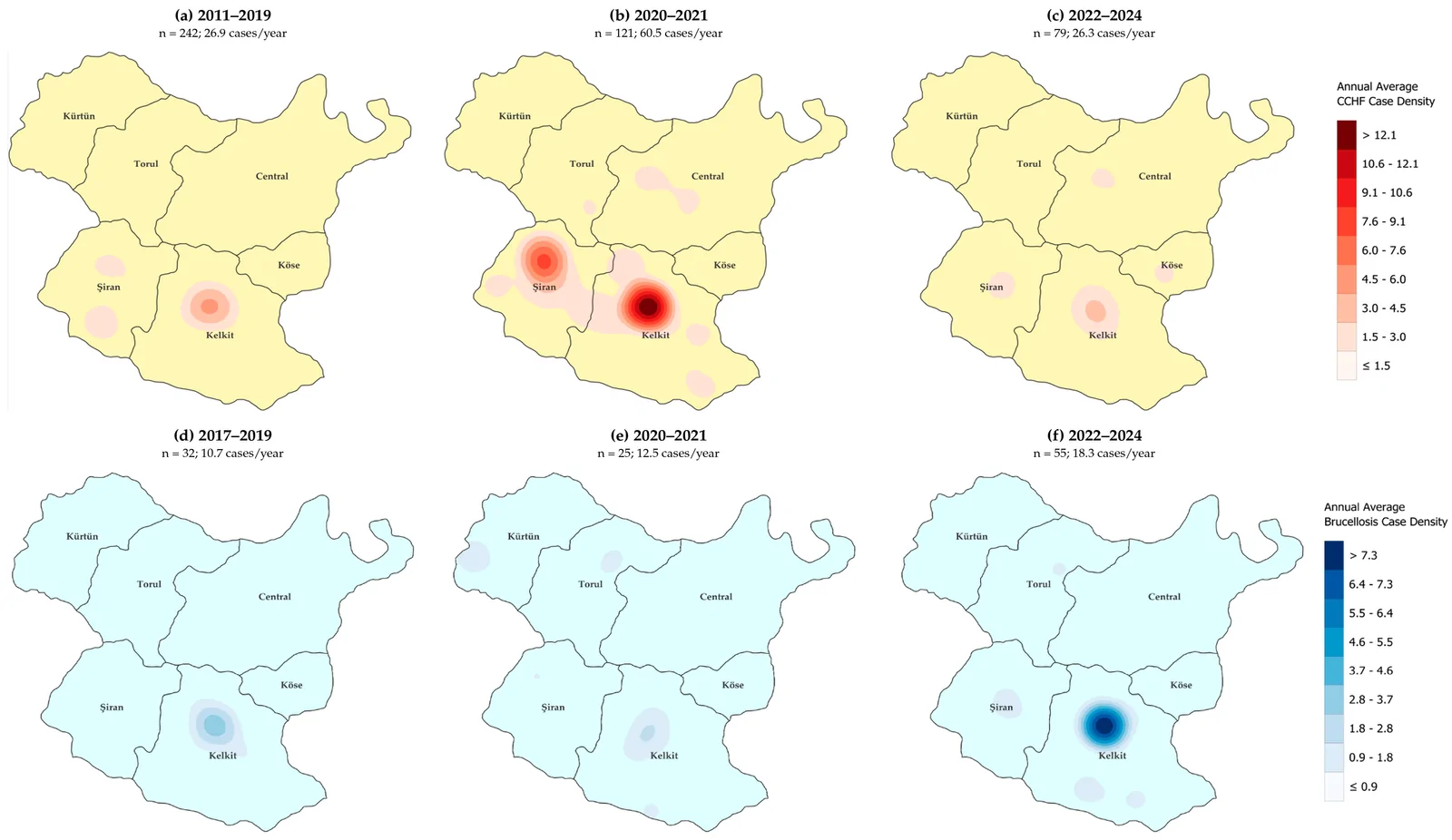
Temporal variation in annualized kernel density patterns of CCHF and brucellosis in Gümüşhane Province. The upper row shows the annualized CCHF density during (**a**) 2011–2019, (**b**) 2020–2021, and (**c**) 2022–2024; the lower row shows the corresponding brucellosis density during (**d**) 2017–2019, (**e**) 2020–2021, and (**f**) 2022–2024. Settlement-level case counts were divided by the number of years in each period before kernel density estimation. All surfaces were generated using a quartic kernel, a 10 km bandwidth, and a 500 m cell size. A common density scale was applied across the three periods within each disease, whereas disease-specific scales were used for CCHF and brucellosis. The maps show annual average case concentration rather than population-adjusted risk.

Annualized disease-specific KDE surfaces showed temporal changes in the magnitude and geographic extent of case concentration (Figure 4). For CCHF, Kelkit remained the principal concentration area during 2011–2019, 2020–2021, and 2022–2024. Annualized density increased during 2020–2021 compared with 2011–2019, with additional concentrations extending toward Şiran and parts of the central and neighboring districts. During 2022–2024, the magnitude and spatial extent of these concentrations decreased, although Kelkit remained the principal concentration area.

Brucellosis displayed a more localized temporal pattern. Case concentration was relatively limited during 2017–2019, became more apparent during 2020–2021, and was most prominent during 2022–2024, particularly in Kelkit. Although the KDE surfaces indicated broad geographic overlap between the two diseases, especially in the southern part of the province, the 10 km smoothing bandwidth did not establish whether this overlap persisted at the settlement level. Sensitivity analyses using alternative bandwidths of 5 and 15 km did not materially change the locations of the principal high-density areas.

### 3.6. Within-Disease Space–Time Interaction

The Knox test demonstrated significant space–time interaction for CCHF at all evaluated spatial and temporal thresholds. The strongest interaction was observed within 5 km and 30 days (observed/expected [O/E] ratio = 1.29, *p* = 0.002). Significant interaction persisted at 10 km and 30 days (O/E = 1.22, *p* < 0.001), 5 km and 60 days (O/E = 1.22, *p* = 0.004), and 10 km and 60 days (O/E = 1.16, *p* = 0.001).

For brucellosis, unadjusted exploratory evidence of space–time interaction was observed only at the narrowest threshold of 5 km and 30 days (O/E = 1.51, *p* = 0.020). No evidence of interaction was observed at 10 km and 30 days (*p* = 0.114), 5 km and 60 days (*p* = 0.077), or 10 km and 60 days (*p* = 0.159).

In the sensitivity analysis excluding within-settlement pairs, the strength of the associations was attenuated. Evidence of between-settlement interaction persisted for CCHF at 10 km and 30 days (O/E = 1.12; *p* = 0.024), whereas the elevated O/E ratio for brucellosis at 5 km and 30 days no longer reached conventional statistical significance (O/E = 1.40; *p* = 0.058).

### 3.7. Spatial and Space–Time Co-Occurrence of CCHF and Brucellosis

Co-occurrence analyses were restricted to the common observation period of 2017–2024 and included 256 CCHF cases and 112 brucellosis cases with usable settlement coordinates.

At the settlement level, the two diseases co-occurred substantially less frequently than expected under random disease labeling. The observed number of cross-disease case pairs assigned to the same settlement was 155, compared with an expected count of 414.8 (O/E = 0.37; lower-tail permutation *p* < 0.001). This pattern persisted when the number of cases contributed by each settlement–disease combination was capped at five, thereby limiting the influence of settlements with large case counts (113 observed vs. 152.4 expected pairs; O/E = 0.74; lower-tail permutation *p* = 0.001). The settlement-level separation also persisted in the urbanicity-conditioned random-labeling analysis, in which disease labels were permuted separately within urban and rural strata. The observed number of same-settlement cross-disease pairs remained 155, compared with 443.0 expected pairs under the conditioned permutation distribution (O/E = 0.35; lower-tail permutation *p* < 0.001). Overall, although both diseases were endemic within the same region, they were recorded in the same settlements substantially less frequently than expected under random labeling.

The cross-disease Knox analysis showed no evidence of excess space–time co-occurrence between CCHF and brucellosis at any threshold combination (Table 3). Observed cross-disease pair counts were consistently lower than expected (O/E = 0.68–0.80), with unadjusted lower-tail permutation *p*-values ranging from 0.025 to 0.041. Lower-than-expected pair counts should not be interpreted as evidence of ecological exclusion or a negative interaction between the two diseases.

## 4. Discussion

This study demonstrates that co-endemic zoonoses occurring within the same livestock-dependent region do not necessarily share a common community-level epidemiological pattern. CCHF and brucellosis accounted for more than 90% of the reported zoonotic disease burden and showed partially overlapping broad areas of case concentration, particularly in and around Kelkit. However, this regional overlap diminished at finer spatial and temporal scales. The diseases co-occurred in the same settlements substantially less frequently than expected under random labeling, and no excess cross-disease space–time co-occurrence was detected during the common observation period. Their temporal profiles also differed markedly, with CCHF displaying a narrow seasonal pattern and brucellosis occurring throughout the year. These findings indicate that geographic co-endemicity should not be assumed to represent shared settlement-level distribution, simultaneous occurrence, or identical public health needs.

### 4.1. Scale-Dependent Spatial Patterns

CCHF and brucellosis showed partially overlapping areas of concentration within the livestock-dependent landscape of southern Gümüşhane. Kelkit was the principal concentration area for both diseases, while Şiran represented a secondary concentration area, particularly for CCHF. This broad geographic pattern is consistent with studies linking CCHF to tick ecology, climate, habitat fragmentation, and livestock-related land use [11,12,13,14], and brucellosis to livestock-intensive regions [15].

However, the apparent overlap diminished at the settlement level. Cross-disease case pairs occurred within the same settlements substantially less frequently than expected under random labeling. The direction of this finding persisted after limiting each settlement and disease combination to a maximum of five cases, indicating that it was not driven solely by settlements with high case counts. Thus, although the diseases shared a broad endemic region, they affected the same villages or neighborhoods considerably less frequently than expected. Importantly, the settlement-level separation persisted after conditioning the random-labeling procedure on urban–rural classification, indicating that the finding was not solely attributable to differences in settlement urbanicity between the two diseases.

This difference reflects the scale of spatial analysis. KDE identifies broad areas of concentration by smoothing cases across a specified bandwidth but cannot determine whether cases originated from the same settlement. Accordingly, the KDE and random-labeling results are complementary: the diseases shared a regional concentration around Kelkit but showed separation at the settlement level.

The distinction is epidemiologically plausible. CCHF is shaped by tick ecology, outdoor activity, animal handling, and the use of agricultural environments. Brucellosis is associated with infected animals, reproductive materials, and unpasteurized dairy products that may be transported beyond their place of production [6,7]. These different exposure pathways may produce distinct local patterns within the same livestock-dependent region. However, because individual exposure histories and food- and animal-movement data were unavailable, these explanations remain hypotheses.

The differences in spatial and settlement-level patterns are further discussed with respect to the demographic and epidemiological characteristics of the cases. CCHF and brucellosis cases had comparable age distributions but differed in sex distribution, settlement urbanicity, and temporal occurrence. CCHF cases were mainly rural and had strong seasonality, with almost all cases occurring between April and September. This pattern is consistent with the known contribution of seasonal tick activity, outdoor exposure, and livestock-related activities to CCHF transmission. In contrast, brucellosis cases had a higher proportion of males and a relatively greater urban representation than CCHF cases, while occurring throughout the year. These findings are compatible with more continuous livestock-related exposure and with the potential extension of brucellosis risk beyond production areas through the distribution and consumption of unpasteurized dairy products. Together, these demographic and temporal differences provide plausible context for the distinct settlement-level patterns observed in the random-labeling and Knox analyses. However, occupation, individual animal contact, food-consumption practices, and mobility were not available in the surveillance dataset; therefore, these mechanisms should be regarded as plausible explanations rather than directly demonstrated determinants of the observed spatial separation.

### 4.2. Temporal and Spatiotemporal Dynamics

The temporal patterns also differed markedly. Almost all CCHF cases occurred between April and September, with peaks in June and July, consistent with the recognized seasonality of CCHF and Hyalomma tick activity in Türkiye [5,11,12,13,14]. Brucellosis occurred throughout the year in the present study, consistent with Turkish case series reporting cases across different seasons, although often with spring–summer predominance [19,20]. Its broader temporal distribution may be related to continuing livestock- and food-related exposure, together with its variable incubation period and insidious clinical presentation [7,19].

Within-disease Knox analyses showed that CCHF exhibited significant spatiotemporal interaction across all evaluated distance and time combinations, whereas the brucellosis association was limited to 5 km and 30 days. The associations were attenuated when within-settlement pairs were excluded, indicating that repeated cases from the same settlements contributed substantially to the primary findings. Nevertheless, limited between-settlement interaction remained detectable for CCHF. Because common settlement-center coordinates were used, these findings indicate settlement-level recurrence and should not be interpreted as individual-level clustering, common exposure, or direct transmission [21].

The cross-disease analysis showed no evidence of excess spatiotemporal co-occurrence between CCHF and brucellosis during the common observation period of 2017–2024. This finding should not be interpreted as evidence of ecological exclusion or a negative biological interaction between the diseases, despite consistently lower observed than expected counts of cross-disease pairs. Rather, the pattern may reflect differences in transmission pathways, seasonal timing, distribution of settlements, and disease-specific detection and reporting processes. CCHF is strongly seasonal and is more closely associated with tick exposure and outdoor or livestock-related activities, whereas brucellosis occurs throughout the year and may be associated with direct animal contact or foodborne exposure. These distinct exposure pathways may reduce the likelihood that cases of the two diseases occur within the same short spatial and temporal windows, even within the same endemic region. In addition, the lower-tail results were based on four nested threshold combinations without adjustment for multiple comparisons and should therefore be regarded as exploratory.

The temporal patterns of the two diseases around the COVID-19 pandemic followed different trajectories, highlighting how a population-wide disruption may affect co-endemic zoonoses in different ways. Annualized CCHF concentration increased from 26.9 cases per year in 2011–2019 to 60.5 in 2020–2021 and then declined to 26.3 in 2022–2024. In contrast, brucellosis showed a more gradual increase, from 10.7 cases per year in 2017–2019 to 12.5 in 2020–2021 and 18.3 in 2022–2024. These contrasting trends may be related to differences in pandemic-associated changes in exposure patterns, healthcare access, and disease recognition [22,23,24]. Because individual mobility, exposure behavior, and healthcare utilization were not directly measured, these observations should be regarded as hypothesis-generating rather than evidence of a causal pandemic effect. More broadly, they suggest that public health emergencies may differentially influence the exposure and detection of co-endemic zoonotic diseases.

### 4.3. Implications for Surveillance and Public Health Practice

The findings support an integrated surveillance approach that combines shared regional infrastructure with disease-specific local action. Coordination between human, animal, and environmental health services provides an appropriate framework for monitoring co-endemic zoonoses [1,2]. Provincial surveillance systems and regional planning resources may be shared, while the timing, location, and content of preventive activities should be tailored to each disease. A broad surveillance system also maintains the visibility of less frequently reported zoonoses, including tularemia and cystic echinococcosis.

Broad geographic priority areas may be shared across co-endemic zoonoses, but settlement-level priorities should be defined separately for each disease. CCHF prevention should be intensified before and during peak tick activity in settlements with recurrent seasonal cases. Brucellosis requires year-round animal-health surveillance, food-safety measures, and education regarding unpasteurized dairy products. The contrasting pandemic-period trajectories further suggest that emergency responses directed at one disease may indirectly alter the exposure, diagnosis, or notification patterns of other diseases. Routine zoonotic surveillance should therefore be maintained during major public health emergencies.

More generally, spatial surveillance should extend beyond static maps. Geographic findings should be interpreted alongside temporal variation, human behavior, animal movements, vector activity, food-distribution networks, healthcare utilization, and diagnostic capacity. Broad regional planning should be complemented by settlement-specific, season-specific, and disease-specific interventions. This approach would preserve the efficiencies of integrated surveillance without assuming that co-endemic zoonoses share identical community-level patterns.

A further consideration when comparing the two diseases is that aside from differences in transmission pathways, there are differences in the chances that cases will be detected and reported. CCHF is usually an acute, potentially severe febrile illness and in an endemic setting such as Gümüşhane, may raise a relatively high clinical suspicion and diagnostic investigation. Brucellosis, in contrast, may follow a more subacute or nonspecific clinical course and is recognized as being susceptible to delayed diagnosis and underreporting [7,15]. Thus, although both diseases are captured within the same national notification framework, differences in clinical presentation, healthcare-seeking behavior, level of suspicion, diagnostic investigation, and reporting may result in differential case ascertainment. Such asymmetry could have influenced the observed spatial and temporal comparisons, including the apparent degree of settlement-level co-occurrence. Accordingly, the divergent patterns identified in this study should be interpreted as differences in recorded human disease occurrence rather than as direct estimates of the underlying incidence or transmission intensity of the two infections.

Although invasive species were not directly evaluated in this study, ecological changes affecting host–environment interactions may represent additional factors influencing zoonotic risk and warrant further investigation within a One Health framework.

### 4.4. Strengths and Limitations

The principal strength of this study is the direct comparison of CCHF and brucellosis within the same province, population, and surveillance system. Gümüşhane provides an informative comparative setting because the two diseases coexist within a livestock-dependent geography but differ in their transmission routes, seasonal characteristics, and expected patterns of community exposure. Examining them within the same epidemiological and reporting environment reduced variation arising from differences in data sources, surveillance structures, and regional context. This design allowed the study to address a question that cannot be answered by separate disease maps: whether geographic co-endemicity translates into shared spatial and temporal patterns at the community level. By demonstrating that partial regional overlap can coexist with settlement-level separation and divergent temporal dynamics, the study provides empirical evidence that geographic co-endemicity is scale-dependent rather than a uniform epidemiological condition. It also offers a transferable multiscale framework for comparing co-endemic zoonoses and determining when broad geographic overlap should be investigated using settlement-level and temporal analyses. These findings have relevance for surveillance policy and resource allocation. They indicate a community-level divergence in disease occurrence: although the two zoonoses are co-endemic and may share regional infrastructure and intersectoral coordination, their spatial and temporal patterns differ at the community level, and the geographic and temporal targeting of interventions should therefore remain disease-specific. However, this divergence is inferred from human case data alone; a comprehensive One Health characterization of the underlying transmission dynamics would require the integration of animal, vector, environmental, and behavioral data, which were beyond the scope of the present surveillance-based study. Village- and neighborhood-level geocoding enabled broad case concentration to be distinguished from settlement-level and spatiotemporal co-occurrence. The analytical framework also builds on the research team’s previous application of GIS to routine surveillance records for leptospirosis in northeastern Türkiye [25], extending a single-disease mapping approach to the comparative analysis of two co-endemic zoonoses. The robustness of the principal findings was examined through multiple sensitivity analyses.

This study has several limitations. As a retrospective analysis of routine surveillance records, it may have been affected by underreporting, diagnostic delay, recording errors, and changes in case definitions or reporting practices over time. Importantly, these sources of ascertainment bias may not have affected the two diseases equally. The acute and potentially severe presentation of CCHF may increase clinical suspicion, diagnostic testing, and notification, whereas the more subacute or nonspecific presentation of brucellosis may make it more susceptible to delayed diagnosis and underdetection. The analyses could not account for this differential ascertainment because individual-level information on symptom onset, healthcare utilization, diagnostic pathways, and testing practices was unavailable. Temporal analyses also relied on the date of diagnosis rather than the date of symptom onset, which was not consistently recorded. This distinction is particularly relevant to the cross-disease comparison because CCHF and brucellosis differ substantially in their incubation periods and clinical course. The relatively short incubation period and acute presentation of CCHF may result in a closer temporal correspondence between symptom onset and diagnosis, whereas brucellosis, which has a longer and more variable incubation period, may present insidiously, and may be diagnosed considerably later after exposure. Consequently, cases acquired within the same community and broadly similar exposure period could receive temporally separated diagnosis dates, particularly when brucellosis diagnosis is delayed. This differential temporal misclassification could have obscured some true space–time co-occurrence between the two diseases and influenced the cross-disease Knox findings. Accordingly, the absence of excess cross-disease co-occurrence should be interpreted cautiously and should not be taken as evidence that the diseases cannot share overlapping exposure periods. Analyses based on symptom-onset dates, or ideally estimated exposure periods where available, would allow for a more accurate assessment of cross-disease temporal co-occurrence.

The concentration of most brucellosis records in the later years limited the assessment of long-term trends. Individual-level information on occupation, tick exposure, animal contact, consumption of unpasteurized dairy products, clinical severity, and laboratory findings was unavailable. Therefore, the mechanisms underlying the observed patterns could not be assessed directly. In particular, the present study drew exclusively on human case notifications and did not incorporate animal, vector, behavioral, or environmental data. Consequently, the divergent patterns observed here should be interpreted as community-level differences in human disease occurrence rather than as a comprehensive One Health characterization of transmission. A fuller One Health assessment would require the integration of data on animal infection and movement, vector abundance and activity, husbandry practices, food-related exposures, and other relevant behavioral and environmental factors.

Cases were geocoded to village or neighborhood centers rather than individual residences, and recorded residence may not represent the actual place of exposure. Assigning identical coordinates to cases from the same settlement may have inflated short-distance interaction in the primary Knox analysis. Excluding within-settlement pairs attenuated the associations, indicating that repeated cases assigned to common settlement centers contributed to the primary findings. Because individual residential coordinates were unavailable, true close-range exposure could not be distinguished from proximity introduced by centroid assignment. The Knox findings should therefore be interpreted as settlement-level interaction rather than as evidence of individual-level clustering, a common exposure source, or direct transmission.

Village- and neighborhood-level population denominators were unavailable; therefore, the point and KDE maps represent case concentration rather than population-adjusted risk. Although alternative KDE bandwidths did not materially change the principal high-density areas, smoothing may have obscured finer-scale differences. Cross-disease analyses were restricted to the common period of 2017–2024, which excluded earlier CCHF cases and reduced statistical power. The settlement-level random-labeling analysis may also have been influenced by settlements with large case counts, although the direction of the finding was preserved in the capped-case sensitivity analysis. Cross-Knox *p*-values were unadjusted and should be interpreted cautiously. The absence of excess cross-disease space–time co-occurrence should not be interpreted as evidence of independence. Finally, the study was conducted in a single province, and its ecological findings may not be directly generalizable or interpreted as individual-level causal relationships.

## 5. Conclusions

CCHF and brucellosis were co-endemic within the same livestock-dependent region but did not exhibit a fully shared community-level epidemiological pattern. Although their broad areas of case concentration partially overlapped, the diseases co-occurred in the same settlements substantially less frequently than expected. Their temporal patterns also differed markedly: CCHF was strongly seasonal, whereas brucellosis occurred throughout the year. Cross-disease analysis showed no evidence of excess space–time co-occurrence during the common observation period.

Broad geographic overlap alone is insufficient to determine whether co-endemic zoonoses affect the same communities or require identical intervention strategies. Regional surveillance infrastructure and intersectoral coordination may be shared across diseases; however, prevention activities should be tailored to disease-specific transmission pathways, settlements, periods of risk, and potentially exposed populations. Because the present analysis was based on human case data, these conclusions describe patterns of human disease occurrence rather than the full One Health transmission system; integration of animal, vector, environmental, and behavioral data will be necessary to clarify the mechanisms underlying these patterns. Broad-scale case maps should therefore be complemented by settlement-level and spatiotemporal analyses before common geographic targets are defined.

## Figures and Tables

**Table 1:** Zoonotic diseases recorded in Gümüşhane Province, 2011–2024.

Disease	*n*	%	First Recorded Year	Age, Median (IQR), Years	Male, *n* (%)
CCHF	442	72.3	2011	49.0 (36–60)	259 (58.6)
Brucellosis	116	19.0	2015	49.5 (38–59)	82 (70.7)
Tularemia	29	4.7	2012	42.0 (21–54)	14 (48.3)
Cystic echinococcosis	20	3.3	2017	32.5 (18–55)	14 (70.0)
Lyme disease	2	0.3	2018	25.0	0 (0.0)
Anthrax	1	0.2	2023	39.0	0 (0.0)
Leptospirosis	1	0.2	2023	41.0	0 (0.0)
Total	611	100.0	—	—	—

CCHF: Crimean–Congo hemorrhagic fever; IQR: interquartile range, Note: The first recorded year may reflect differences in the availability or completeness of disease-specific surveillance records and should not be interpreted as the year of disease emergence.

**Table 2:** District-level distribution and notification rates of CCHF and brucellosis.

District	CCHF, *n*	CCHF Rate	Brucellosis, *n*	Brucellosis Rate
Kelkit	185	29.9	76	21.2
Şiran	132	45.8	11	6.7
Central	59	8.0	10	2.3
Torul	41	24.5	8	8.4
Köse	23	21.7	2	3.3
Kürtün	2	1.1	7	6.7
Total	442	21.0	114	9.3

Note: Rates are expressed per 100,000 person-years using annual district populations. CCHF rates cover 2011–2024 (442 cases), whereas brucellosis rates cover 2017–2024 (114 cases). Two brucellosis cases recorded in 2015 were outside the period used for district-level brucellosis rate calculations. Annual district populations were obtained from the Address-Based Population Registration System of the Turkish Statistical Institute.

**Table 3:** Cross-disease Knox space–time co-occurrence between CCHF and brucellosis during 2017–2024.

Spatial Threshold	Temporal Threshold	Observed Pairs	Expected Pairs	O/E	Upper-Tail *p*	Lower-Tail *p*
≤5 km	≤30 days	42	61.6	0.68	0.968	0.041
≤10 km	≤30 days	80	109.5	0.73	0.979	0.025
≤5 km	≤60 days	82	114.2	0.72	0.978	0.026
≤10 km	≤60 days	162	203.2	0.80	0.970	0.034

Note: Cross-disease pairs comprised one CCHF case and one brucellosis case during 2017–2024 (CCHF, *n* = 256; brucellosis, *n* = 112). Expected counts were obtained by independently permuting diagnosis dates among fixed case locations within each disease 9999 times using a fixed random seed of 2026. Calendar dates were used, and pairs occurring exactly at the specified spatial or temporal thresholds were included. Upper-tail *p*-values evaluated excess space–time co-occurrence, whereas lower-tail *p*-values evaluated fewer-than-expected pairs. Monte Carlo *p*-values were calculated using the +1 correction. The table presents unadjusted exploratory *p*-values. Distances were calculated using WGS 84/UTM Zone 37N. O/E: observed-to-expected ratio; CCHF: Crimean–Congo hemorrhagic fever.

## Data Availability

Restrictions apply to the availability of these data. The data were obtained from the Gümüşhane Provincial Health Directorate and are not publicly available due to patient confidentiality and institutional data-protection requirements. Access to the data is subject to permission from the Republic of Türkiye Ministry of Health.

## Abbreviations

The following abbreviations are used in this manuscript:

CCHF	Crimean–Congo hemorrhagic fever
DEGURBA	Degree of urbanization
GHS-SMOD	Global human settlement layer settlement model
GIS	Geographic information system
IQR	Interquartile range
KDE	Kernel density estimation
O/E	Observed-to-expected ratio
UTM	Universal Transverse Mercator
WGS 84	World Geodetic System 1984
